# Interplay between TGF-β signaling and long non-coding RNAs in digestive system cancers: mechanisms and biological implications

**DOI:** 10.1007/s13577-025-01341-5

**Published:** 2025-12-23

**Authors:** Penghui Li, Di Huang, Xinyu Gu

**Affiliations:** 1 Department of Gastrointestinal Surgery, The First Affiliated Hospital, College of Clinical Medicine, Henan University of Science and Technology, Luoyang, 471000 Henan China; 2 Department of Child Health Care, The Third Affiliated Hospital of Zhengzhou University, Zhengzhou, 450052 Henan China; 3 Department of Oncology, The First Affiliated Hospital, College of Clinical Medicine, Henan University of Science and Technology, No. 24 Jinghua Road, Jianxi District, Luoyang, 471000 Henan China

**Keywords:** TGFβ signaling, Long non-coding RNAs, Digestive system cancers, Clinical features, Biomarkers

## Abstract

The interaction between the transforming growth factor beta (TGFβ) signaling pathway and long non-coding RNAs (lncRNAs) has been known to contribute to the progression and metastasis of digestive system cancers. Dysregulated expression of lncRNAs associated with the TGFβ-Smad signaling pathway is correlated with several clinical features in digestive system cancers. These lncRNAs regulate multiple biological functions, including epithelial-to-mesenchymal transition (EMT), tumor growth, and immune responses. They interact with key molecules in the TGFβ-Smad pathway to influence gene transcription and cellular behavior. Alterations in the expression of these lncRNAs can serve as valuable biomarkers for early detection and prognosis. Targeting the lncRNA-TGFβ axis offers a novel approach to cancer treatment. This review summarizes the interactions between the TGFβ signaling pathway and lncRNAs in digestive system cancers, highlighting their potential in diagnosis, prognosis, and therapy.

## Background

Tumors of the digestive system, including cancers of the colon, stomach, pancreas, liver, and esophagus, are major contributors to global morbidity and mortality rates worldwide [1–3]. The prognosis for digestive system cancers remains poor due to challenges in early diagnosis and effective treatment. The underlying mechanisms are complex, involving genetic, environmental, and lifestyle factors. Consequently, there is a pressing need to understand the molecular and cellular mechanisms to improve diagnostic and therapeutic outcomes.

The Transforming growth factor-beta (TGFβ) pathway has been implicated in digestive system tumors, where it can function both as a tumor suppressor in early stages and a tumor promoter in advanced stages [4, 5]. Initially, TGFβ inhibits cellular proliferation and induces apoptosis, serving as a barrier to early cancer development. However, with tumor progression, tumor cells develop mechanisms to evade these suppressive effects, thereby turning TGFβ into a facilitator of tumor growth, invasion, and metastasis [6–8]. This is particularly evident in digestive system cancers, where TGFβ affects the tumor microenvironment by promoting epithelial-to-mesenchymal transition (EMT), enhancing cell motility, and modulating the immune response to provide an environment conducive to cancer progression [9]. It is imperative to understand the complex functions of TGFβ for developing targeted therapies to block its tumor-promoting activities without disrupting its tumor-suppressive functions.

Long non-coding RNAs (lncRNAs) have increasingly been recognized as vital regulators in numerous cancers, including digestive system cancers, by modulating gene expression and cellular pathways [10–12]. Specifically, lncRNAs significantly interact with the TGFβ pathway, a critical axis in cell proliferation, differentiation, and apoptosis. These interactions are fundamental to the progression and metastasis of digestive system tumors. Recent studies have reported several lncRNAs that regulate the TGFβ-Smad pathway, thereby influencing tumor behavior and the dynamics of the tumor microenvironment. Such findings underscore the potential of lncRNAs as therapeutic targets and diagnostic markers. In this review, we summarize the latest research progress on the molecular mechanisms and functional roles of lncRNAs associated with the TGFβ pathway in tumorigenesis and tumor progression. We believe that this discussion will not only highlight the biological significance of lncRNAs but also emphasize their utility in developing novel cancer therapies.

### Overview of the TGFβ pathway

#### Composition and activation of the TGFβ pathway

**Fig. 1:**
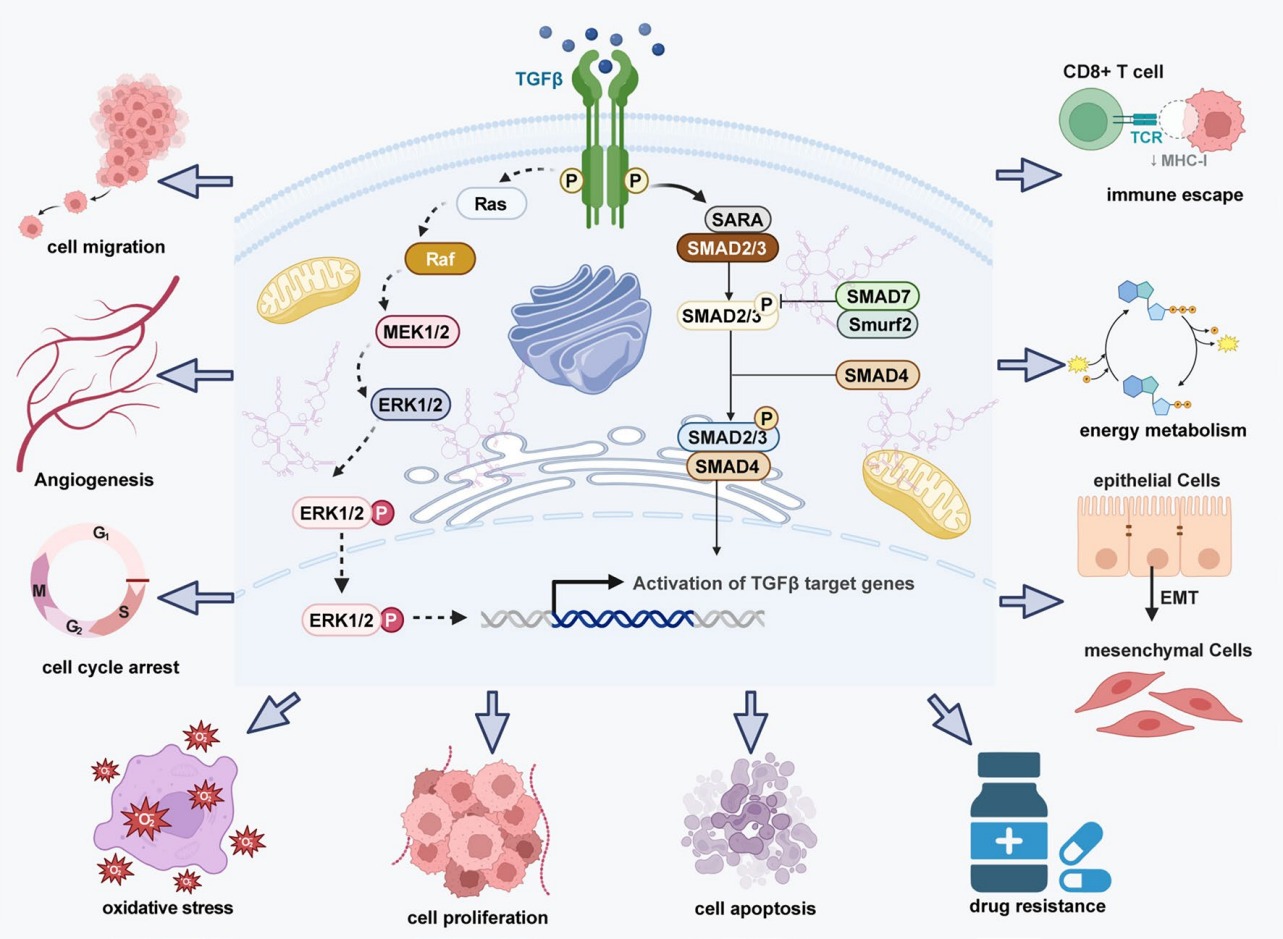
Overview of the TGFβ Signaling Pathway. The core components and activation mechanism of the TGFβ signaling pathway, as well as its diverse roles are demonstrated in cancer progression. The pathway is initiated by the binding of TGFβ ligands to type II receptors (TGFβRII), which then recruit and phosphorylate type I receptors (TGFβRI). Activated receptors phosphorylate receptor-regulated SMADs (R-SMADs: SMAD2/3), which form a complex with co-SMAD (SMAD4) and translocate to the nucleus to regulate gene expression. In parallel, TGFβ signaling can activate non-canonical pathways, such as the Ras-ERK cascade, influencing cell cycle and proliferation. Downstream effects include regulation of migration, angiogenesis, cell cycle arrest, apoptosis, oxidative stress, drug resistance, immune evasion, energy metabolism, and epithelial–mesenchymal transition (EMT)

Under physiological conditions, the TGFβ signaling pathway constitutes a complex network involving multiple ligands, receptors, and SMAD proteins (Fig. 1) [4, 13–15]. These components have been implicated in cell growth, differentiation, and the maintenance of homeostasis. TGFβ ligands initiate signal transduction by binding to type II and type I serine/threonine kinase receptors. These receptors subsequently phosphorylate receptor-regulated SMAD proteins (R-SMADs), such as SMAD2 and SMAD3, which form complexes with co-mediator SMAD (SMAD4) and accumulate in the nucleus to regulate transcription of target genes. The activation and regulation of this signaling pathway are intricately controlled through different mechanisms to ensure precise cellular responses [16, 17]. For instance, the phosphorylation of R-SMADs constitutes a key step in TGFβ signal transduction, triggering the translocation of SMAD proteins to the nucleus and subsequent regulation of gene expression [18]. In addition, inhibitory SMADs negatively regulate signal transmission by competing with TGFβ receptors or promoting their ubiquitination and degradation, thereby creating a negative feedback loop that modulates signal strength and duration [19]. Furthermore, SMAD proteins can interact with other transcription factors or transcriptional coactivators such as CBP (CREB-binding protein). These interactions enable the SMAD complex to bind more effectively to DNA, thereby promoting the expression of specific genes. For example, SMAD2 and SMAD3 can enhance their transcriptional activity by binding with co-activators such as CBP, to induce the expression of TGFβ target genes such as those encoding extracellular matrix proteins and cell cycle regulators [20]. In addition, environmental factors such as mechanical stress, inflammatory factors, and changes in the extracellular matrix can significantly affect the activity and effects of TGFβ signaling. For example, during tissue repair, inflammatory factors released by damaged tissue can activate the TGFβ signaling, promoting fibroblast activation and extracellular matrix deposition, crucial for wound healing [21]. Mechanical stress regulates the signaling pathway by enhancing the activity of TGFβ receptors or altering cell responsiveness to TGFβ, thus impacting cell behavior and function [22]. In all, the TGFβ signaling pathway ensures the proper execution of cellular functions and the maintenance of tissue homeostasis through different levels of regulatory mechanisms [23]. The TGFβ family comprises three isoforms, TGFβ1, TGFβ2, and TGFβ3, with TGFβ1 being the most extensively studied in digestive system malignancies. In this review, references to specific isoforms (TGFβ1 or TGFβ2) reflect the particular focus of the cited studies. While these isoforms signal through overlapping SMAD-dependent mechanisms, their expression patterns and functional relevance may vary by cancer type [24].

#### Complex role of TGFβ in digestive system cancers

The TGFβ pathway plays a pivotal yet paradoxical role in cancer, particularly in digestive system malignancies such as gastric and colorectal cancers. Notably, it exhibits stage-dependent functionality, acting as a tumor suppressor in the early phases of tumorigenesis, and as a tumor promoter during advanced disease progression.

In the early stages of cancer, TGFβ predominantly functions as a tumor suppressor. It enforces cell cycle arrest at the G1 phase, thereby preventing the replication of damaged DNA and serving as a critical checkpoint against malignant transformation [25]. Additionally, TGFβ promotes apoptosis in pre-malignant cells and maintains epithelial cell differentiation, thus preserving tissue architecture and preventing dedifferentiation into more stem-like, tumor-prone phenotypes [26, 27].

The tumor-suppressive effects of TGFβ are also evidenced by genetic studies in murine models. While isolated deletion of core pathway components such as TGFBRII or SMAD4 rarely leads to spontaneous tumorigenesis, their loss significantly accelerates tumor progression when combined with oncogenic mutations such as KRAS or APC. This has been observed in several digestive organs, including the pancreas, intestine, liver, and esophagus. These findings suggest that TGFβ signaling does not primarily prevent tumor initiation but rather acts as a barrier to early oncogenic progression under conditions of cellular stress or injury. [25]. Notably, a few lncRNAs such as Linc-pint, MBNL1-AS1, and NKILA may function as tumor suppressors by cooperating with TGFβ signaling during early tumorigenesis, although direct stage-specific evidence remains limited and warrants further investigation.

In contrast, during the advanced stages of cancer, TGFβ signaling becomes reprogrammed to support malignancy. It induces epithelial–mesenchymal transition (EMT), a cellular reprogramming process that imparts tumor cells with increased motility, invasiveness, and resistance to apoptosis. Beyond direct effects on tumor cells, TGFβ also exerts immunosuppressive functions by inhibiting cytotoxic T lymphocytes and natural killer (NK) cell activity, thereby facilitating immune evasion [28]. Moreover, it contributes to the remodeling of the tumor microenvironment through promotion of angiogenesis and extracellular matrix (ECM) degradation, further supporting tumor invasion and metastasis [28]. However, further studies are needed to clearly delineate the stage-specific expression and functions of TGFβ-related lncRNAs in early versus late digestive system cancers, as current data remain limited and fragmented.

Therapeutic strategies targeting the TGFβ signaling pathway remain challenging due to its context-dependent dual roles in cancer. Therefore, the design of effective interventions must selectively inhibit the tumor-promoting functions of the pathway without disrupting its physiological tumor-suppressive roles [29].

## Overview of lncRNAs

### Functional impact of long non-coding RNAs (lncRNAs) in cellular regulation

Long non-coding RNAs (lncRNAs) are RNA molecules that exceed 200 nucleotides in length. Because they lack open reading frames, they do not encode proteins [30–32]. Transcribed by RNA polymerase II, lncRNAs participate in numerous regulatory mechanisms within cells, influencing gene expression at multiple levels—from chromatin modification to transcriptional and post-transcriptional processes [33–36]. LncRNAs can be categorized into several types depending on their genomic location relative to protein-coding genes, including intergenic lncRNAs, intronic lncRNAs, overlapping lncRNAs, and antisense lncRNAs [37]. They interact with several chromatin-modifying proteins to alter chromatin states and modulate transcriptional activity at specific genomic loci [38]. LncRNAs can either enhance or suppress the transcription of neighboring or distant genes by binding transcription factors or components of the transcription machinery [39]. Furthermore, lncRNAs influence splicing, mRNA stability, and translation by interacting with other RNAs and proteins that process them. Physiologically, lncRNAs contribute to cell differentiation and development. In addition, they regulate different aspects of the immune system, impacting both innate and adaptive immune responses [40, 41]. They often interact with the cytoskeleton or organelles to maintain cellular structure and function.

### Regulatory role of lncRNAs in digestive system cancers

Long non-coding RNAs (lncRNAs) are key regulators in the development and progression of digestive system cancers [42–44]. LncRNAs impact the epigenetic patterns of cancer cells by interacting with chromatin-modifying proteins, thereby altering gene expression patterns crucial for cancer progression. For instance, lncRNAs can recruit the polycomb repressive complex to specific genomic sites to silence tumor suppressor genes or activate oncogenes [45]. They interact with different signaling molecules and pathways essential in cancer biology [46–48]. They function as competitive endogenous RNAs (ceRNAs) or molecular sponges to sequester miRNAs, thus regulating the efficacy of miRNA-targeted mRNAs and affecting signaling pathways controlling cell proliferation, apoptosis, and metastasis [49–51]. LncRNAs are involved in reshaping the tumor microenvironment, a critical factor in cancer progression [40, 52, 53]. They influence the behavior of stromal cells such as fibroblasts and immune cells, thereby promoting an environment conducive to tumor growth and invasion. LncRNAs regulate immune responses and create an immunosuppressive environment favorable for tumor survival and growth. Moreover, lncRNAs contribute to the metastatic potential of cancer cells by regulating EMT [54–56]. Altogether, lncRNAs hold promise as biomarkers for the early detection, prognosis, and monitoring of therapeutic responses in digestive system cancers due to their cell type specificity and stability in body fluids.

### Crucial interplay between TGFβ pathway and long non-coding RNAs in digestive system cancers

The interaction between the TGFβ pathway and long non-coding RNAs (lncRNAs) contributes to the progression of digestive system cancers [57]. LncRNAs are key regulatory factors interacting with the TGFβ pathway to modulate cellular processes such as EMT, cell proliferation, and metastasis, which are crucial for the initiation and development of cancer. The TGFβ pathway is well-known for inducing EMT, a process that enhances the migratory and invasive capabilities of cancer cells. For instance, the lncRNA ELIT-1 promotes EMT by enhancing the TGFβ-Smad signaling pathway. This interaction augments the migratory and invasive potential of cancer cells, thereby facilitating metastasis of pancreatic cancer. In addition, lncRNAs regulate the progression of digestive system tumors at the molecular level through multiple pathways affecting the TGFβ pathway. Several recent studies have significantly advanced our understanding of the intricate molecular mechanisms and critical functional roles of lncRNAs in association with the TGFβ pathway in digestive system cancers. These studies underscore the complex interplay between lncRNAs and the TGFβ signaling pathway, highlighting how lncRNAs influence key processes such as cell differentiation, proliferation, and migration through this pathway. Although insights into how lncRNAs modulate the TGFβ signaling components offer promising avenues for novel therapeutic strategies to mitigate the tumorigenic potential and inhibit the progression of digestive system cancers.

#### Esophageal cancer

Several lncRNAs have been identified as oncogenic regulators in esophageal squamous cell carcinoma (ESCC), enhancing tumor progression through their interaction with the TGFβ signaling pathway (Table 1). For instance, SNHG17 and LINC00239 are significantly upregulated in esophageal cancer tissues and are further induced by TGFβ1 treatment in vitro [58, 59]. Their expression levels positively correlate with advanced TNM stage, deeper tumor invasion, and increased lymph node metastasis (Table 2). Functionally, both lncRNAs promote cancer cell proliferation, migration, invasion, and EMT. Mechanistically, SNHG17 enhances c-Myc expression by binding to c-Jun and activating the PI3K/AKT pathway, while LINC00239 facilitates c-Myc transcription by modulating MBP-1 binding to the c-Myc promoter. These actions collectively contribute to aggressive tumor behavior.

**Table 1:** Expression patterns and clinical relevance of lncRNA-TGFβ axis in digestive system tumors

Cancer types	LncRNAs	Expression	Clinical features	Refs
Esophageal cancer	NKILA	Downregulated	Tumor stages, lymph node metastasis, T stage, and clinical stage	[90]
Esophageal cancer	SNHG17	Upregulated	TNM stage, tumor depth of invasion, tumor differentiation, lymph node metastasis, and overall survival	[27]
Esophageal cancer	NCK1-AS1	Upregulated	Overall survival	[84]
Esophageal cancer	LINC00239	Upregulated	TNM stage, tumor depth of invasion, lymph node metastasis, and overall survival	[89]
Gastric cancer	FBXO18-AS	Upregulated	Overall survival	[28]
Gastric cancer	SND1-IT1	Upregulated	Tumor depth of invasion, lymph node metastasis, and TNM stage	[29]
Gastric cancer	XIST	Upregulated		[48]
Gastric cancer	LINC00665	Upregulated	Tumor depth of invasion, lymph node metastasis, and TNM stage, overall survival, and disease-free survival	[35]
Gastric cancer	NR027113	Upregulated	Lymph node metastasis, distant metastasis, and overall survival	[32]
Gastric cancer	MBNL1-AS1	Downregulated	Overall survival	[50]
Liver cancer	snaR	Upregulated	Tumor metastasis	[43]
Liver cancer	lncRNA-ATB	Upregulated	HBV infection, and TNM stage	[51]
Liver cancer	UCA1	Upregulated	Tumor grade, and overall survival	[3]
Liver cancer	LINC01980	Upregulated	Tumor grade, stage, and overall survival	[77]
Liver cancer	TP73-AS1	Upregulated	Overall survival	[78]
Liver cancer	lncRNA-ATB	Upregulated	liver cirrhosis, microvascular invasion, macrovascular invasion, encapsulation, and overall survival	[73]
Colorectal cancer	HOXC-AS3	Downregulated	Overall survival	[38]
Colorectal cancer	MIR503HG	Downregulated	Overall survival	[74]
Colorectal cancer	TP73-AS1	Upregulated	Overall survival	[36]
Colorectal cancer	SNHG6	Upregulated	Overall survival	[53]
Colorectal cancer	VPS9D1-AS1	Upregulated	Lymph node metastasis and TNM stage, and overall survival	[41]
Colorectal cancer	LINC00941	Upregulated	Lymph node metastasis, American Joint Committee on Cancer (AJCC) status, and overall survival	[79]
Pancreatic cancer	Linc-pint	Upregulated	Tumor size	[75]

**Table 2:** Biological functions and mechanisms regulated by the lncRNA-TGFβ axis in digestive system tumors

Cancer types	LncRNAs	Role	Functions	Mechanism	Refs
Esophageal cancer	NKILA	Tumor suppression	Cell migration, and invasion	TGF-β, NF-κB, MMP14, and IκBα	[90]
Esophageal cancer	MALAT1	Tumor progression	EMT	TGF-β	[68]
Esophageal cancer	SNHG17	Tumor progression	Cell proliferation, cell apoptosis, cell migration, cell invasion, and EMT	TGF-β1, c-Myc, c-Jun, PI3K, and AKT	[27]
Esophageal cancer	NCK1-AS1	Tumor progression	Cell migration, and invasion	TGF-β1	[84]
Esophageal cancer	LINC00239	Tumor progression	Cell proliferation, cell migration, cell invasion, and EMT	c-Myc, and MBP-1	[89]
Gastric cancer	FBXO18-AS	Tumor progression	Cell proliferation, cell migration, cell invasion, and EMT	TGF-β1, and Smad	[28]
Gastric cancer	SND1-IT1	Tumor progression	Cell proliferation, cell migration, cell invasion, and EMT	miR-124, COL4A1, and TGF-β1	[29]
Gastric cancer	XIST	Tumor progression	Cell proliferation, cell migration and invasion, apoptosis	miR-185, and TGF-β1	[48]
Gastric cancer	LINC00665	Tumor progression	Cell proliferation, cell migration, cell invasion, apoptosis, cell cycle arrest, and EMT	TGF-β	[35]
Gastric cancer	NR027113	Tumor progression	Cell migration, cell invasion, and EMT	TGF-β	[32]
Gastric cancer	MBNL1-AS1	Tumor suppression	Cell proliferation, cell apoptosis, cell migration, and cell invasion	miR-424-5p, TGF-β, and SMAD	[50]
Liver cancer	snaR	Tumor progression	Cell migration, and invasion	TGF-β	[43]
Liver cancer	lncRNA-ATB	Tumor progression	Autophagy, cell migration, and invasion	TGF-β	[51]
Liver cancer	UCA1	Tumor progression	Cell proliferation, and cell metabolism	TGF-β1, UCA1, and HXK2	[3]
Liver cancer	LINC01980	Tumor progression	Cell migration, cell invasion, EMT, and lung cancer	TGF-β, SMAD, LINC01980, miR-376b-5p, and E2F5	[77]
Liver cancer	lncRNA SBF2-AS1	Tumor progression	Cell proliferation, cell migration, and cell invasion	miR-361-5p, and TGF-β1	[22]
Liver cancer	TP73-AS1	Tumor progression	M2 macrophage polarization	miR-539, MMP-8, and TGF-β1	[78]
Liver cancer	lncRNA-ATB	Tumor Progression	EMT, cell invasion, and distant organ colonization	miR-200, ZEB1, ZEB2, IL-11, and STAT3	[73]
Colorectal cancer	HOXC-AS3	Tumor suppression	Cell migration, and invasion	miR-1269, and TGF-β2	[38]
Colorectal cancer	MIR503HG	Tumor suppression	Cell migration, and invasion	miR-153, and TGF-β2	[74]
Colorectal cancer	TP73-AS1	Tumor progression	Cell migration, and invasion	TGF-β1	[36]
Colorectal cancer	SNHG6	Tumor progression	Cell proliferation, cell migration, and cell invasion	UPF1, miR-101-3p, ZEB1, TGF-β, and Smad	[53]
Colorectal cancer	VPS9D1-AS1	Tumor progression	Infiltration of CD8^+^T cells and tumor growth	TGF-β, and ISG	[41]
Colorectal cancer	LINC00941	Tumor progression	Cell migration, cell invasion, and EMT	SMAD4/β, TrCP, TGF-β, and SMAD2/3	[79]
Colorectal cancer	TUG1	Tumor progression	Cell migration, cell invasion, and EMT, and lung cancer	TGF-β, TWIST1, and EMT	[8]
Pancreatic cancer	XIST	Tumor progression	Cell proliferation, cell migration, and cell invasion	miR-141-5p, and TGF-β2	[65]
Pancreatic cancer	PVT1	Tumor progression	Autophagy, cell migration, and invasion	PVT1, TGF-β, and Smad	[82]
Pancreatic cancer	XIST	Tumor progression	Cell proliferation, cell invasion, and apoptosis	miR-34a, YAP, EGFR, and TGF-β1	[66]
Pancreatic cancer	LINC01638	Tumor progression	Cell migration, and invasion	TGF-β1	[85]
Pancreatic cancer	Linc-pint	Tumor progression	Cell proliferation	TGF-β1	[75]

Similarly, MALAT1 is another oncogenic lncRNA that positively correlates with TGFβ1 expression both in vivo and in vitro [60]. Knockdown of MALAT1 effectively inhibits TGFβ1-induced EMT, highlighting its role as a downstream effector of TGFβ-mediated tumor progression. Moreover, NCK1-AS1 is upregulated in ESCC and associated with poor patient prognosis. It enhances TGFβ1 expression and promotes cancer cell migration and invasion. Notably, these effects can be reversed by TGFβ pathway inhibition, suggesting that NCK1-AS1 acts at least partially through TGFβ signaling [61]. Conversely, TGFβ inhibitors can reduce the effects of NCK1-AS1 overexpression, indicating its potential therapeutic significance.

In contrast, certain lncRNAs act as tumor suppressors in ESCC by counteracting tumor-promoting pathways. NKILA (NF-κB Interacting LncRNA) is significantly downregulated in ESCC tissues and its low expression is negatively correlated with tumor stage, lymph node metastasis, and clinical severity [62]. Functionally, NKILA inhibits cancer cell migration and invasion both in vitro and in vivo. Mechanistically, it suppresses the phosphorylation of IκBα and subsequent activation of NF-κB, thereby reducing the expression of MMP14, a matrix metalloproteinase associated with invasiveness. Although NKILA does not directly regulate canonical TGFβ-Smad signaling, its inhibition of NF-κB signaling may interact with or counterbalance TGFβ-induced EMT and inflammatory responses, reflecting a broader antagonism to tumor-promoting signaling cascades [62].

#### Gastric cancer

Several lncRNAs have been identified to promote the progression of gastric cancer (GC) by enhancing TGFβ signaling or its downstream oncogenic effects. High expression levels of FBXO18-AS, SND1-IT1, XIST, LINC00665, and NR027113 have been consistently observed in GC tissues [63–67]. Clinically, SND1-IT1 and LINC00665 are positively associated with greater tumor invasion depth, lymph node metastasis, and higher TNM stage, whereas elevated levels of FBXO18-AS, LINC00665, and NR027113 are indicative of poor prognosis.

Functionally, FBXO18-AS and LINC00665 promote gastric cancer cell proliferation, migration, invasion, and EMT [63, 66, 67]. Notably, LINC00665 has been validated as an independent prognostic biomarker through both univariate and multivariate analyses [66]. Silencing LINC00665 results in G0/G1 cell cycle arrest in GC cells, suggesting its role in tumor growth regulation.

Mechanistically, these oncogenic lncRNAs are closely linked to TGFβ signaling. SND1-IT1 facilitates TGFβ1-induced EMT by acting as a sponge for miR-124, thus releasing repression of EMT-promoting genes [64]. XIST, another oncogenic lncRNA, represses miR-185, leading to increased TGFβ1 expression and promotion of GC cell migration and invasion [65]. Functionally, NR027113 has been reported to cooperate with TGFβ signaling to enhance the malignant progression of gastric cancer, further supporting its role as an oncogenic lncRNA [63].

**Fig. 2:**
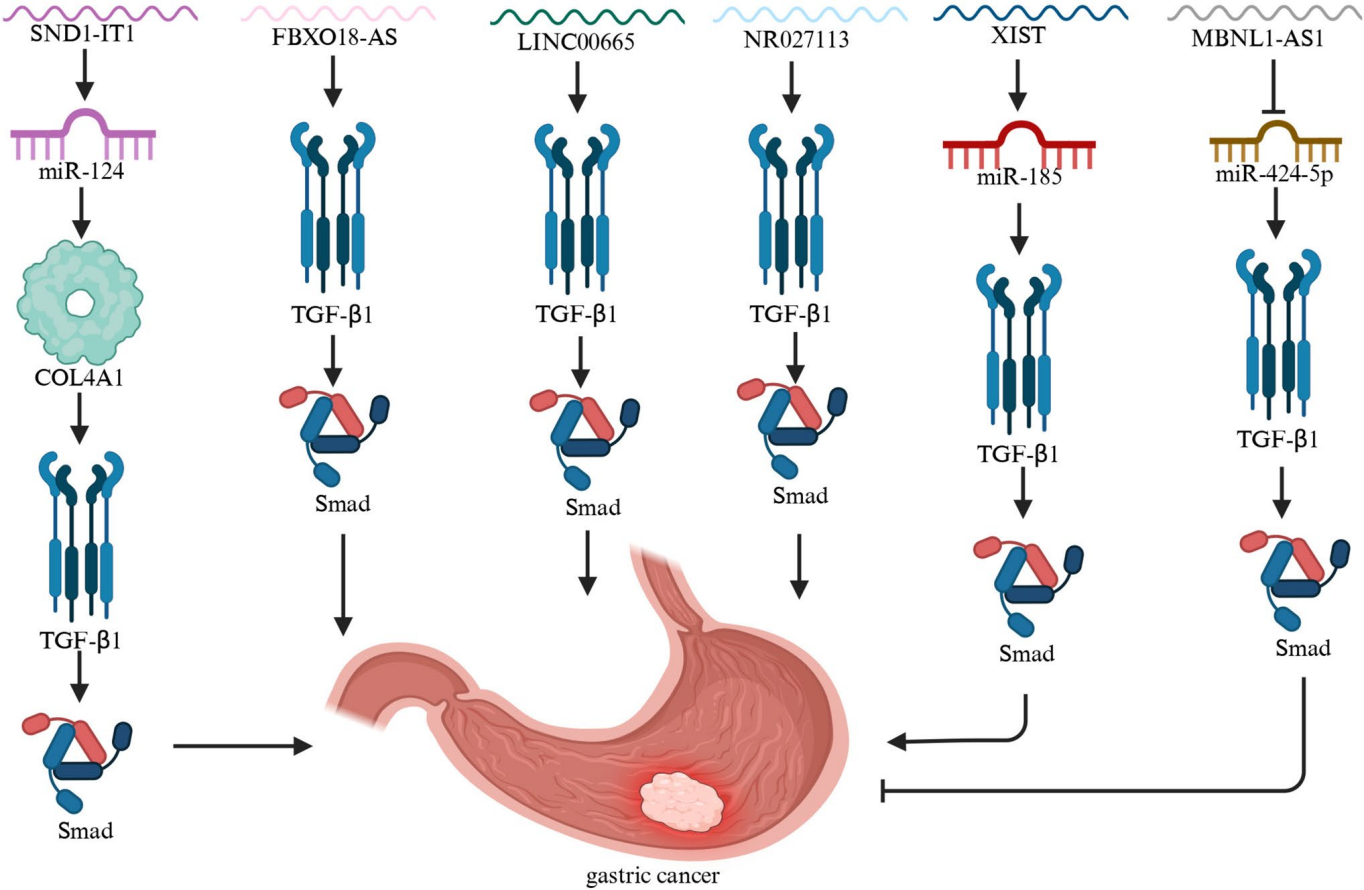
Mechanisms of the lncRNA-TGFβ Axis in Gastric Cancer. SND1-IT1 promotes TGFβ1-induced EMT by sponging miR-124. Other lncRNAs, including FBXO18-AS, LINC00665, and NR027113, enhance gastric cancer progression through interactions with the TGFβ pathway. Silencing of XIST upregulates miR-185, leading to reduced TGFβ1 expression and tumor suppression. MBNL1-AS1 acts as a tumor suppressor by targeting miR-424-5p and regulating Smad7, thereby inhibiting TGFβ/Smad-mediated EMT and metastasis. Activation pathways are indicated by arrows, while inhibitory interactions are marked with blunt-ended lines

Conversely, a subset of lncRNAs plays tumor-suppressive roles by interfering with TGFβ signaling or its downstream oncogenic effects. MBNL1-AS1 is notably downregulated in GC tissues, and its reduced expression correlates with poor prognosis [68]. Functionally, MBNL1-AS1 inhibits tumor proliferation and metastasis. Mechanistically, it acts by sponging miR-424-5p, which in turn regulates SMAD7, a negative regulator of the TGFβ/SMAD pathway. Through this axis, MBNL1-AS1 effectively suppresses TGFβ-mediated signaling, thereby inhibiting EMT and tumor progression (Fig. 2).

#### Liver cancer

In hepatocellular carcinoma (HCC), numerous lncRNAs are upregulated and act as oncogenes by promoting TGFβ signaling or facilitating downstream pro-tumorigenic events. Notably, snaR, LINC01980, SBF2-AS1, lncRNA-ATB, TP73-AS1, and UCA1 are consistently overexpressed in HCC tissues and cell lines [69–75]. snaR promotes HCC cell migration and invasion by upregulating TGFβ1 expression. This effect is reversible upon TGFβ1 inhibition, indicating that snaR exerts its oncogenic role via TGFβ pathway activation [72]. LncRNA-ATB, a well-studied TGFβ-induced transcript, facilitates metastasis by sponging miR-200 family members, which leads to upregulation of zinc-finger E-box-binding homeobox 1 (ZEB1) and ZEB2, key transcription factors of EMT. It also enhances IL-11 expression and activates the STAT3 pathway, promoting distant organ colonization [74]. LINC01980 is associated with advanced clinical stages and poor prognosis. It promotes EMT and cellular invasion by targeting miR-376b-5p, thereby increasing E2F5 expression [71]. Silencing LINC01980 significantly suppresses lung metastasis in vivo. SBF2-AS1 enhances tumor progression by sponging miR-361-5p, leading to derepression of TGFβ1 and promoting cancer cell proliferation and migration [73]. UCA1 promotes tumor energy metabolism by increasing TGFβ1 expression, which enhances glucose uptake, lactate production, and ATP generation, supporting tumor cell survival and growth [70]. TP73-AS1 regulates macrophage polarization and extracellular matrix remodeling by targeting miR-539 and modulating MMP-8 expression, contributing to a pro-tumor microenvironment [69]. Collectively, these oncogenic lncRNAs reinforce TGFβ-associated signaling to drive EMT, metastasis, immune evasion, and metabolic reprogramming in HCC.

Current literature on tumor-suppressive lncRNAs in HCC that antagonize TGFβ-mediated tumor progression remains sparse. Most identified lncRNAs in HCC promote malignancy via direct or indirect enhancement of TGFβ signaling.

#### Colorectal cancer

In colorectal cancer (CRC), several lncRNAs act as oncogenes by promoting tumor growth, immune evasion, or metastasis through modulation of the TGFβ signaling pathway. These include TP73-AS1, SNHG6, VPS9D1-AS1, LINC00941, and TUG1, all of which are significantly upregulated in CRC tissues compared to normal controls [76–79].

**Fig. 3:**
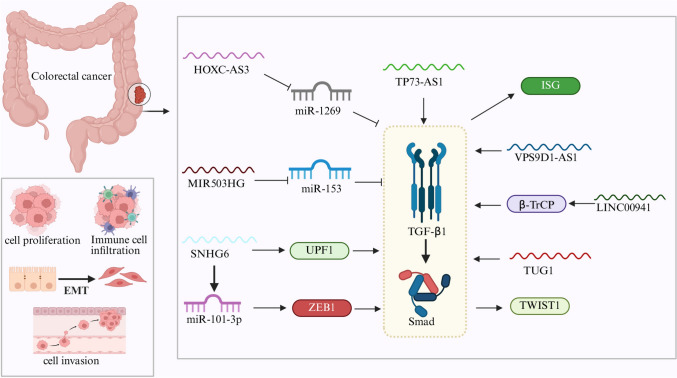
Mechanisms of the lncRNA-TGFβ Axis in Colorectal Cancer. HOXC-AS3 inhibits TGFβ2 signaling by sponging miR-1269, while MIR503HG suppresses tumor progression via the miR-153-TGFβ2 axis. Conversely, SNHG6 downregulates UPF1, leading to ZEB1 upregulation via the TGFβ/Smad pathway and promoting EMT. TP73-AS1 enhances TGFβ1 expression, facilitating cancer progression. VPS9D1-AS1 supports immune evasion and tumor growth through the TGFβ–ISG axis. LINC00941 promotes EMT by binding the SMAD4 MH2 domain and blocking its degradation via β-TrCP, thereby activating SMAD2/3 signaling. TUG1 contributes to metastasis through the TGFβ–TWIST1-EMT pathway. Activation pathways are indicated by arrows, while inhibitory interactions are marked with blunt-ended lines

TP73-AS1 promotes CRC cell invasion and migration by upregulating TGFβ1 expression. This effect can be reversed by TGFβ pathway inhibitors, suggesting direct involvement in TGFβ-mediated tumor promotion [76]. SNHG6 facilitates EMT by downregulating Up-frameshift 1 (UPF1), a key mRNA surveillance factor, which in turn leads to increased ZEB1 expression through activation of the TGFβ–Smad signaling cascade [77]. VPS9D1-AS1 promotes tumor growth and immune escape by activating the TGFβ-interferon-stimulated gene (ISG) axis. It reduces the expression of IFNAR1 on CD8 + T cells, thereby impairing their cytotoxic function. Antisense oligonucleotide targeting of VPS9D1-AS1 significantly suppresses tumor growth in vivo [79]. LINC00941 contributes to EMT and metastasis by directly binding the MH2 domain of SMAD4, thereby preventing its ubiquitination by β-TrCP and stabilizing the TGFβ/SMAD2/3 signaling complex [78]. Clinically, high LINC00941 levels are associated with lymph node metastasis and advanced AJCC stages. TUG1 acts through the TGFβ-TWIST1-EMT axis to enhance metastatic potential in CRC [80]. These findings highlight how oncogenic lncRNAs mechanistically link to various branches of the TGFβ signaling pathway to drive colorectal cancer progression (Fig. 3).

In contrast, several lncRNAs function as tumor suppressors by negatively regulating the TGFβ signaling cascade. Notably, HOXC-AS3 and MIR503HG are downregulated in CRC tissues, and their reduced expression is associated with poor patient prognosis [81, 82]. HOXC-AS3 inhibits CRC progression by sponging miR-1269, thereby derepressing TGFβ2 inhibition and blocking EMT. This results in reduced tumor cell migration and invasion [82]. MIR503HG exerts its suppressive effects via the miR-153/TGFβ2 pathway, downregulating TGFβ2 expression and suppressing the metastatic potential of CRC cells [81]. Together, these tumor-suppressive lncRNAs counteract the oncogenic activation of TGFβ signaling, providing a potential therapeutic avenue for CRC intervention.

#### Pancreatic cancer

Several lncRNAs, such as XIST, PVT1, and LINC01638, are significantly upregulated in pancreatic cancer (PC) tissues compared to adjacent normal tissues, and are associated with enhanced tumor proliferation, migration, and invasion [83–85]. XIST contributes to tumor progression by regulating two TGFβ-related pathways. It promotes EMT via the miR-141-5p/TGFβ2 axis, and also enhances TGFβ1-induced EMT by modulating the miR-34a-YAP-EGFR signaling cascade [83–85]. The knockdown of PVT1 reduces the expression of mesenchymal markers such as Snail, Slug, β-catenin, N-cadherin, and vimentin, whereas it increases that of the epithelial marker E-cadherin. XIST promotes the progression of PC through the miR-141-5p/TGFβ2 axis [84, 86]. Both pathways lead to increased cell motility and invasion. PVT1 activates the TGFβ-Smad signaling pathway, leading to upregulation of mesenchymal markers such as Snail, Slug, β-catenin, N-cadherin, and vimentin, while downregulating the epithelial marker E-cadherin. This contributes to EMT and promotes aggressive tumor behavior [85]. LINC01638, also upregulated in PC and in patient plasma, enhances tumor growth and invasion, though its direct mechanistic link to TGFβ remains less defined. However, experimental data show that TGFβ1 treatment reverses the inhibitory effects of silencing this lncRNA, suggesting a TGFβ-mediated regulatory loop [87]. Collectively, these lncRNAs act as oncogenes by interacting with or enhancing TGFβ signaling pathways, driving PC progression.

In contrast to the oncogenic lncRNAs, Linc-pint functions as a tumor suppressor in pancreatic cancer. Linc-pint is significantly downregulated in both PC tissues and plasma of patients. Its expression positively correlates with TGFβ1 levels in patients with pancreatic ductal adenocarcinoma (PDAC) [87]. Functionally, overexpression of Linc-pint suppresses PDAC cell proliferation, and this tumor-inhibitory effect is synergistically enhanced by TGFβ1. Importantly, treatment with TGFβ pathway inhibitors diminishes the suppressive effect of Linc-pint, suggesting that it mediates its anti-tumor activity at least in part through TGFβ signaling. Thus, Linc-pint may represent a context-specific lncRNA that cooperates with TGFβ signaling to reinforce its tumor-suppressive role during early tumorigenesis.

### Clinical applications of TGFβ and lncrna interactions in digestive system cancers

Despite technological advancements, cancer diagnosis and treatment remain challenging, underscoring the need to identify novel tumor biomarkers [88–90]. The interaction between the TGFβ signaling pathway and lncRNAs has been shown to influence various aspects of tumor biology and may serve as a valuable resource for clinical applications in digestive system malignancies, including diagnosis, prognosis, and therapeutic intervention.

### Diagnosis and prognosis

Many lncRNAs that modulate TGFβ signaling pathways have been found to correlate with tumor stage, metastasis, and overall survival in digestive system cancers. These associations provide a basis for their use as diagnostic or prognostic biomarkers. For instance, SNHG17 and LINC00239 are upregulated and positively correlate with tumor TNM stage, invasion depth, and lymph node metastasis in ESCC [58, 59]. Univariate and multivariate Cox analyses have further validated SNHG17 as an independent prognostic factor [59]. Similarly, high expression of **FBXO18-AS**, **LINC00665**, and **NR027113** has been linked to poor prognosis in GC [63, 66, 67]. Notably, LINC00665 has been independently associated with patient outcomes in multivariate analysis [66]. In HCC, circulating levels of lncRNAs such as snaR and UCA1, are elevated compared to healthy controls, suggesting utility in non-invasive early detection [70, 72]. Furthermore, LINC01638 and Linc-pint have shown diagnostic and prognostic relevance based on plasma expression levels in PC [83, 87]. Taken together, these lncRNAs demonstrate strong potential to serve as biomarkers for patient stratification, early diagnosis, and prognostic prediction across different types of digestive system cancers.

### Therapeutic targets

Despite rapid advancements in treatment methods, cancer therapy remains one of the most challenging issues worldwide [88, 91]. The lncRNA/TGFβ axis also holds promise as a therapeutic target, given its involvement in key oncogenic processes such as EMT, immune evasion, and metastasis. A number of lncRNAs discussed in previous sections, such as SNHG17, MALAT1, XIST, SBF2-AS1, NKILA, and Linc-pint, have been shown to regulate tumor behavior in preclinical studies through their interaction with the TGFβ pathway [59, 60, 62, 65, 73]. Importantly, modulating the expression of these lncRNAs can significantly influence cancer cell proliferation, migration, invasion, and treatment resistance [87]. These findings highlight their potential as molecular targets for therapeutic intervention across multiple digestive system malignancies. Various therapeutic strategies are currently being explored to target the lncRNA/TGFβ axis in digestive system cancers. These include RNA interference techniques, such as small interfering RNAs (siRNAs) and antisense oligonucleotides (ASOs), designed to downregulate oncogenic lncRNAs; restoration of tumor-suppressive lncRNAs to re-establish negative regulation of TGFβ signaling; and the use of small-molecule inhibitors or monoclonal antibodies to block TGFβ receptors or interfere with SMAD-mediated transcriptional activation. Additionally, combination therapies that integrate TGFβ pathway inhibitors with conventional treatments, such as chemotherapy, radiotherapy, or immune checkpoint blockade, are being investigated to improve overall therapeutic efficacy. These approaches offer promising avenues for intervention, though further clinical validation is needed to establish their safety and effectiveness.

Future research should prioritize the functional characterization of key regulatory lncRNAs, the elucidation of cancer subtype-specific regulatory networks, and the design of multimodal therapeutic strategies. Advances in single-cell and spatial transcriptomics may further reveal how the lncRNA-GFβ axis functions within the tumor ecosystem. Ultimately, deepening our understanding of this axis holds the potential to transform current paradigms in cancer diagnosis, prognosis, and treatment, paving the way toward more personalized and effective therapies for digestive system cancers.

## Conclusions

Digestive system cancers remain a major global health burden due to late diagnosis, aggressive progression, and limited treatment options. The interplay between the TGFβ signaling pathway and long non-coding RNAs (lncRNAs) plays a pivotal role in tumor growth, metastasis, and immune evasion across multiple digestive malignancies. These shared mechanisms highlight the lncRNA–TGFβ axis as a potential unifying framework for understanding cancer progression.

Targeting this axis offers promising diagnostic, prognostic, and therapeutic opportunities. However, challenges remain in validating key lncRNAs, understanding context-specific effects, and developing precise delivery systems. Future studies should focus on functional characterization of regulatory lncRNAs and integration of emerging technologies such as single-cell and spatial transcriptomics. Advancing our knowledge in this area may enable more personalized and effective treatment strategies for digestive system cancers.

## Data Availability

Not available.
